# Immunologic Signatures of Peripheral Blood T Cells Reveal the Outcome of p53MVA Vaccine and Pembrolizumab Treatment in Patients with Advanced Ovarian Cancer

**DOI:** 10.1158/2767-9764.CRC-23-0394

**Published:** 2023-12-20

**Authors:** Ferdynand J. Kos, Paul Frankel, Mihaela Cristea, Melissa Eng, Raechelle Tinsley, Shannon Dempsey, Nora Ruel, Daphne Stewart, Thanh H. Dellinger, Don J. Diamond

**Affiliations:** 1 Department of Hematology and Hematopoietic Cell Transplantation, Beckman Research Institute, City of Hope, Duarte, California.; 2 Department of Computational and Quantitative Medicine, Beckman Research Institute, Duarte, California.; 3 Department of Medical Oncology, City of Hope National Medical Center, Duarte, California.; 4 Clinical Trials Office, City of Hope National Medical Center, Duarte, California.; 5 Department of Surgery, City of Hope National Medical Center, Duarte, California.

## Abstract

**Purpose:**

Our previous studies indicated that p53-reactive T cells were associated with clinical benefit in patients with advanced ovarian cancer who were treated with p53-expressing modified vaccinia Ankara (p53MVA) vaccine and gemcitabine chemotherapy. To replace chemotherapy with an approach that will enhance vaccine efficacy and antitumor immunity, we treated patients with p53MVA in combination with PD-1 checkpoint blocker, pembrolizumab. We also attempted to further characterize the activation status of T cells prior to vaccination and during treatment.

**Experimental Design:**

Patients received up to three triweekly vaccinations concurrent with pembrolizumab, followed by pembrolizumab monotherapy at 3-week intervals. Correlative studies analyzed peripheral blood T-cell phenotypes and profiles of immune function gene expression.

**Results:**

We observed 6/28 (21%) patients with a clinical benefit to therapy, including 3 partial responses (PR) and 3 patients with stable disease (SD) for 6+ months. The median progression-free survival was 1.8 months (95% confidence interval: 1.7–3.8) and median overall survival was 15.1 months (9.4–30.4). Two patients remain progression-free at 28 and 33 months. Of the 18 patients evaluable in correlative studies, 6 were immunologic responders of whom 5 had clinical benefit (3 PR, 2 SD). Immunologic non-responders expressed in pretreatment peripheral blood mononuclear cell samples high levels of mRNA for multiple molecules associated with terminally differentiated T cells.

**Conclusions:**

p53MVA/pembrolizumab immunotherapy showed promising antitumor activity in patients who demonstrated functionally competent peripheral blood T cells. Detection of markers of terminally differentiated T cells before treatment may identify patients unlikely to respond to p53MVA/pembrolizumab.

**Significance:**

The activity of a combination immunotherapy of p53 vaccine and PD-1 checkpoint blockade in patients with platinum-resistant ovarian cancer was evaluated in a phase II trial. Clinical benefit was correlated with the responsive immune status of patients before and during the treatment, defining potential predictive markers for immune therapy.

## Introduction

Ovarian cancer is a highly heterogeneous and aggressive malignancy. More than 75% of patients are diagnosed with late-stage disease and require extensive surgery followed by platinum-based chemotherapy. Most patients respond well to initial treatment with platinum-based chemotherapy but they frequently develop recurrent disease and chemoresistance. Five-year survival rate for patients with distant metastases is only 30% (1). Therefore, there is a great need to improve ovarian cancer outcomes by new treatment approaches.

Immunotherapy for the treatment of ovarian cancer remains controversial (2). Early observations that intratumoral T-cell infiltration is associated with better survival led to the hypothesis that ovarian cancer is immunogenic and potentially responsive to immunotherapy (3). Challenging this hypothesis is a recent observation that the tumor mutational burden, which correlates with the number of neoantigens, is in the lower end of spectrum for ovarian cancer with only 1–3.5 mutations/Mb (immunogenic melanoma has 14–47 mutations/Mb). Moreover, only around 1% of these mutations are detected by autologous tumor-associated T cells (4). As tumor mutational burden is a good predictor of response to immune checkpoint blockade (ICB; ref. 5), it is not surprising that early trials of ICB-targeting PD-1 receptor have shown only modest monotherapy activity. In the Keynote-100 trial, pembrolizumab demonstrated an 8.0% [95% confidence interval (CI): 5.4–11.2] response rate and a median progression-free survival (PFS) of only 2.1 months in both the less heavily and more heavily pretreated patient cohorts (6). The issue of ovarian cancer immunogenicity and response to immunotherapy is further complicated by the heterogeneity of the tumor microenvironment and its immune cell infiltration patterns including increased immunosuppressive regulatory T cells and tumor-associated macrophages (7).

Somatic *TP53* gene mutations and associated p53 protein overexpression are common in ovarian cancer, particularly in ovarian high-grade serous cancer (*TP53* is altered in >96% of cases; ref. 8). p53 overexpression and its cytosolic localization may lead to increased presentation of p53-derived epitopes. In fact, the majority of epitopes are derived from nonmutated wild-type sequences that can be presented for T-cell recognition on tumor cells (9–13). To activate strong and persistent T-cell immune responses against p53 self-epitopes, treatment must overcome self-tolerance and other resistance mechanisms. Vaccination with p53MVA leads to the delivery of transgene-encoded p53 protein to antigen presenting cells for activation of specific T-cell responses. Activated effector and memory cells can migrate to the tumor site where they can execute their p53-specific antitumor functions (14). Thus, the p53MVA vaccine may increase the magnitude of tumor-specific T cells, and this T cell activity may be amplified by reversing PD-1 inhibition by ICB. ICB sensitivity and PFS are associated with baseline CD8^+^ T-cell clone size and cytotoxicity. Presence of large, cytotoxic clones before treatment has been shown to be prognostically favorable in metastatic melanoma, implying that much of the effect of ICB relies on preexisting CD8^+^ T-cell responses (15). Moreover, clonal replacement of tumor-infiltrating lymphocytes (TIL) by peripheral T cells is a major drive of ICB responses. Expanded peripheral T cells in responding patients infiltrate tumors and replace tumor-specific TIL following PD-1 blockade (16, 17).

On the basis of these observations and our experience with the p53MVA cancer vaccine (11, 12, 14, 18–21), we executed a phase II clinical trial combining p53MVA and pembrolizumab in patients with recurrent ovarian, fallopian tube, or primary peritoneal cancer. The main objective was to assess response rate [complete response (CR) + partial response (PR)], with secondary endpoints including the median PFS, overall survival (OS), clinical benefit [CR+PR+SD (stable disease) >6 months], safety, and tolerability of the combined treatment. As part of a prespecified exploratory objective, we analyzed peripheral blood T-cell responses and profiles of immune function gene expression before and during the treatment. Here we report the findings of this study.

## Materials and Methods

### Patients, Study Design, and Treatment

Patients 18 years or older with histologically confirmed epithelial ovarian, fallopian tube, or primary peritoneal cancer and platinum-resistant recurrence of disease, defined as recurrence of progression within 0–6 months following platinum-based chemotherapy, were eligible to enter the study. Patients required an Eastern Cooperative Oncology Group (ECOG) performance status of 0–2, and measurable disease by RECIST 1.1, or detectable disease defined as cancer antigen 125 (CA-125) at least two times the upper limit of normal value, ascites or pleural effusion attributed to tumor or other lesions that do not meet the definition for target lesions. Up to four prior chemotherapy regimens for recurrent disease were allowed. Additional eligibility criteria included confirmed positivity by either p53 overexpression by IHC defined as >10% of the cells within the tumor staining positive or p53 mutation as determined by mutational analysis. Exclusion criteria included prior treatment with anti-PD-1/PD-L1 antibodies, chronic use of immunosuppressive medications, cardiac disorders, history of noninfectious pneumonitis, active autoimmune disease or active central nervous system disease.

City of Hope (COH) Institutional Review Board (IRB) approved the study and it was registered with ClinicalTrials.gov (NCT03113487). All patients signed written informed consent for participation in this study, including treatment, collection of blood, and data analysis in accordance with the ethical institutional standards and with the Declaration of Helsinki, Belmont Report, and U.S. Common Rule. The correlative blood analysis from three healthy controls (female, age: 39, 60, and 67) were obtained from a COH IRB-approved blood study of cytomegalovirus seropositive healthy volunteers (COH IRB 93140). The primary endpoint of this phase II study was the response rate (CR+PR). Secondary endpoints included median PFS, median OS, and clinical benefit (CR+PR+SD>6 months). Patients received 5.6 × 10^8^ pfu of p53MVA delivered as an intramuscular injection into the upper arm over the deltoid muscle followed by pembrolizumab 200 mg intravenously 30 minutes after vaccination. Both agents were given on day 1 of a 21-day cycle for three cycles, followed by single agent pembrolizumab 200 mg every 3 weeks up to 35 cycles (2 years total), as described previously (19, 21).

### Trial Agents

p53MVA was manufactured using GMP-grade materials at the Center for Biomedicine and Genetics at COH. The final product was diluted in PBS with 7.5% lactose at a concentration of 5.6 × 10^8^ pfu/mL. Merck Sharp & Dohme Corp., a subsidiary of Merck & Co., Inc., provided Keytruda (pembrolizumab) for the study.

### Toxicity Evaluation and Clinical Response Assessment

Patients were observed for 1 hour following each p53MVA and pembrolizumab injections with observation of temperature, blood pressure, oxygen saturation, and cutaneous injection site, and contacted 24–48 hours after each injection to evaluate related complications. Patients were assessed for immune-related adverse events (AE) every 3 weeks, including monitoring for thyroid dysfunction and other checkpoint inhibitor–associated toxicities. Patients were also followed for assessment of disease progression and survival outcome. CT or PET/CT scan was performed at baseline and every 9 weeks (every 3 cycles) while undergoing protocol therapy. AEs were classified using the NCI Common Terminology Criteria for Adverse Events v 5.0. Patients who discontinued study treatment for a reason other than disease progression were assessed every 12 weeks per standard of care. Patients with AEs of grade >1 were followed until resolution of AE (grade 0 or 1) or until the beginning of a new anticancer therapy, whichever occurs first. When feasible, response was confirmed after two consecutive radiographic assessments at least 4 weeks apart. Clinical response was assessed by immune-related response evaluation criteria in solid tumors (irRECIST) (22).

### Correlative Studies and Immunologic Assessment

Peripheral blood samples were collected at study entry and after initiation of therapy at three weekly intervals. Peripheral blood mononuclear cells (PBMC) prepared by Ficoll-Paque gradient separation were cryopreserved until analysis. In the initial studies, the frequency of p53-specific CD137^+^CD8^+^ and CD137^+^CD4^+^ T cells in PBMC samples from all patients and timepoints were analyzed by flow cytometry as described previously (14, 21). Additional flow cytometric analysis was performed to identify the frequency of total and activated T effector (TEF) and T effector memory (TEM) cells in PBMC according to established standards of immunophenotyping (23). PBMC samples were stained with the following antibodies: CD3-FITC, CD4-BV421, CD8-PerCP-Cy5.5, CD45RA-BV510, CD45RO-PE-CF594, CD197-PE, HLA-DR-AF700, CD38-APC (BD Biosciences) and analyzed by flow cytometry (BD FACSCelesta, BD Biosciences). Data acquired in FACSDiva (BD Biosciences) were analyzed in FlowJo (FlowJo LLC). Normal human peripheral blood was obtained from deidentified volunteer blood donors through the COH Blood Donor Center.

Serum CA-125 levels were analyzed every 3 weeks during routine clinical laboratory testing. The method used was Siemens Advia Centaur chemiluminescent immunoassay based on OC 125 and M11 antibodies (Fujirebio Diagnostics, Inc.).

Multiplex gene expression analysis of PBMC samples was performed as described previously (14). Briefly, total RNA was isolated from PBMC samples using miRNeasy mini kit (Qiagen). RNA fragmentation and quality control was determined by 2100 Bioanalyzer (Agilent). All RNA samples were normalized to 20 ng/µL. RNA expression was analyzed by NanoString nCounter platform (NanoString Technologies) by digitally detecting and counting in a single reaction without amplification using nCounter PanCancer Immune Profiling Panel (XT-CSO-HIP1-12). Post-hybridization probe-target mixture was quantified with nCounter Digital Analyzer and all data were analyzed in nSolver 4.0 software package (NanoString).

### Statistical Analysis

This phase II study employed Simon MiniMax two-stage design to have 85% power to detect a true promising response rate of 25%, with a type I error of 8% for declaring a true discouraging response rate of 8% as worthy of further consideration (based on KEYNOTE-100). Specifically, if two or more of the initial 17 patients responded (CR or PR), accrual would continue to a total of 28 patients, where at least 5 clinical responders (R) were required to consider this combination active based on the response rate, although final determination of response was to be based on the totality of the clinical endpoints.

Calculations used SAS 9.4, R version 4.0, and GraphPad Prism v 9.2.0 software. Statistical significance was based on a two-sided test unless otherwise specified and paired and unpaired *t* tests were applied as noted. Survival was evaluated using Kaplan–Meier methods, and study maturity was estimated by summarizing the median follow-up of alive patients. PFS was defined as the time from first dose of the study medication to the first documented disease progression or death due to any cause, which occurs first. If a patient started a new therapy, the patient was censored at that time for PFS.

### Data Availability Statement

The data generated in this study are available from the corresponding author upon reasonable request with the permission of COH. Individual participant data will not be shared; only deidentified data will be made available according applicable laws and regulations.

## Results

### Clinical Responses

**FIGURE 1:**
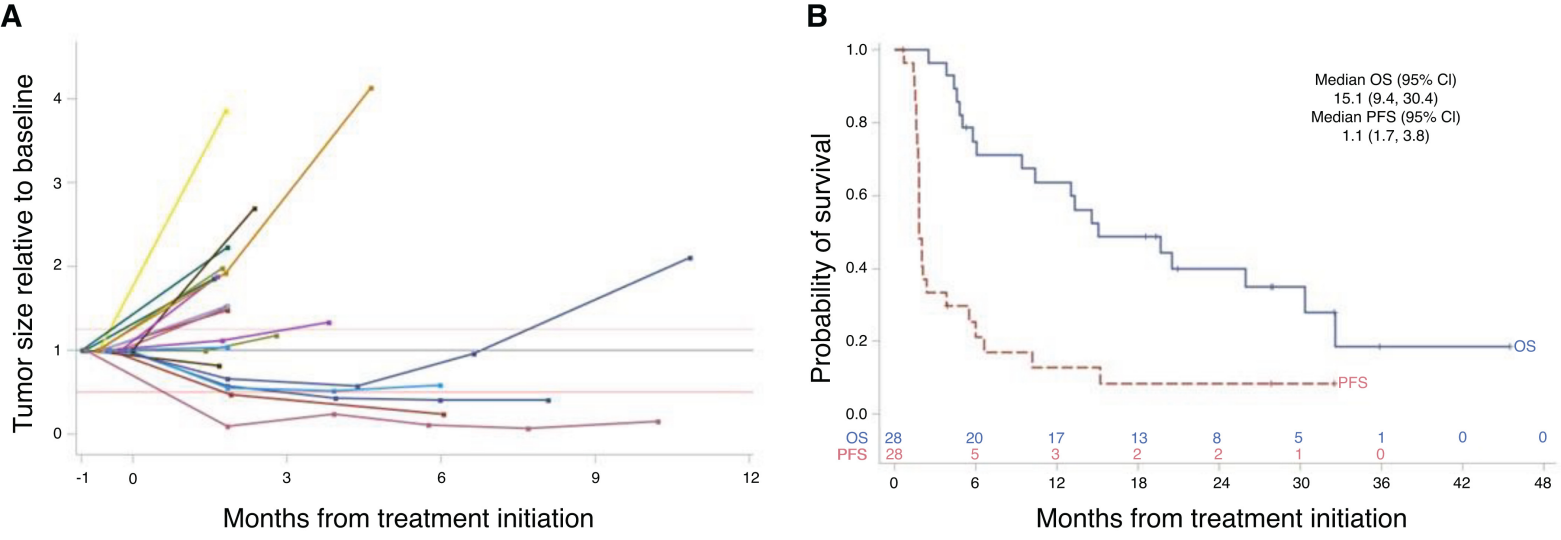
Clinical response of patients with ovarian cancer treated with p53MVA and pembrolizumab. Relative changes in tumor sizes derived from radiological evaluations (**A**) show that 3/28 PR patients had a decrease of 76.4%, 93.3%, and 59.5% in tumor burden (irRECIST). Twenty-one of 28 patients had at least baseline and one subsequent measurement. OS and PFS are shown in **B**. The numbers above the *X*-axis in B show numbers of patients at risk at the given timepoint after the start of treatment for OS and PFS. Two patients remain progression-free at 28 and 33 months.

The trial was initiated in May 2019 and 29 patients were accrued over the course of 3 years (see Supplementary Table S1 for representativeness of study participants). The first patient was removed from the protocol after cycle 1 of p53MVA and pembrolizumab due to sepsis caused by a pelvic abscess. The infection was deemed to have been present at baseline, so the patient was ineligible. The characteristics of the 28 eligible and treated patients are summarized in Table 1 and Table 2. During the first stage of the trial accruing 17 patients, 2 patients achieved radiologically confirmed PRs with reduction of the tumor burden of 59.5% and 76.3% (UPN017 and UPN012). In the second stage of enrollment, one more patient reached the status of clinical PR with a 93.3% reduction in tumor burden (UPN018). Patient UPN017 completed all clinical trial–related treatments and continues on single agent pembrolizumab 33 months later. One patient with SD (UPN020) remains on single agent pembrolizumab 28 months later. Of 28 treated and evaluated patients, 3 achieved PR and 3 patients experienced SD of at least 6 months (including 1 patient with SD for 6 months), for a clinical benefit rate of 21.4% (6/28) patients (Fig. 1A). In all treated patients, the median PFS was 1.8 months (95% CI: 1.7–3.8) and the median OS is 15.1 months (9.4–30.4; Fig. 1B). The median follow-up of alive patients is 28 months.

**TABLE 1:** Patient characteristics

Demographic/disease characteristic	*N* = 28 *n* (%)
Age (years), Median (range)	64.0 (43–79)
Disease site	
Ovary	21 (75%)
Fallopian tube	6 (21%)
Primary peritoneal	1 (4%)
Histology	
High-grade serous carcinoma	28 (100%)
ECOG	
0	13 (46%)
1	15 (54%)
p53 status	
Molecular mutation	24 (86%)
IHC	6 negative (21%) 13 positive (46%) 9 NA (32%)
BRCA status	
Molecular mutation	2 positive (7%) 13 negative (46%) 13 NA (46%)
Prior treatment, Median (range)	
Total drug regimens	4 (2–7)
Total drug regimens for recurrent/refractory disease	2 (1–4)
Chemotherapy regimens recurrent/refractory disease	2 (1–4)
Prior PARP inhibitor	12 (43%)
Prior bevacizumab	20 (71%)

**TABLE 2:** Patient treatment, immune response, disease status, PFS, and OS (ordered by clinical response/PFS)

Patient ID	Age	ECOG	Prior therapies for recurrent disease	Vaccine doses	Immune response	Clinical best response	PFS (months)	OS (months)
UPN017	71	0	2	3	R	PR	32.5+	32.5+
UPN012	54	1	2	3	R	PR	15.2	28.0+
UPN018	55	0	3	3	R	PR	10.2	19.4+
UPN020	69	0	3	1	N/A	SD	27.8+	27.8+
UPN008	70	0	3	3	R	SD	6.6	35.8+
UPN013	64	1	3	3	R	SD	6.0	14.6
UPN019	70	0	2	1	N/A	SD	5.5	18.6+
UPN009	74	1	2	3	R	SD	3.9+	30.4
UPN005	64	0	2	3	NR	SD	3.8	19.7
UPN023	79	1	4	1	N/A	N/A	0.7+	9.4
UPN003	63	0	1	2	N/A	PD	2.4	25.9
UPN007	53	1	3	3	N/A	PD	2.1	3.8
UPN010	55	1	4	3	NR	PD	2.0	6.1
UPN028	67	0	3	3	NR	PD	2.0	5.3+
UPN001	64	1	1	3	NR	PD	1.8	45.5+
UPN026	57	1	3	3	NR	PD	1.8	5.0
UPN016	47	1	1	3	NR	PD	1.8	20.5
UPN015	66	0	2	3	NR	PD	1.8	32.6
UPN021	57	1	4	3	NR	PD	1.8	4.4
UPN025	56	0	2	3	NR	PD	1.8	15.1
UPN022	54	0	2	3	NR	PD	1.7	4.8
UPN027	62	1	1	3	NR	PD	1.7	13.0
UPN014	68	1	1	2	N/A	PD	1.6	10.4
UPN006	43	0	2	2	N/A	PD	1.6	5.8
UPN024	72	1	4	3	NR	PD	1.6	4.6
UPN002	68	0	3	2	N/A	PD	1.5	2.5
UPN011	63	1	2	2	N/A	PD	1.4	13.3
UPN004	68	1	1	1	N/A	PD	0.7	20.9+

Abbreviations: +: censored, N/A: not assessed/not available, NR: immune non-responder, PD: progressive disease, PR: partial response, R: immune responder; SD: stable disease, TE: too early (no CT scans).

p53MVA/pembrolizumab treatment was well tolerated with the most common AE being injection site reaction, fatigue, and flu-like symptoms (Supplementary Table S2). In 4 patients, we observed grade 3 AEs at least possibly related to treatment, one per patient, consisting of elevation in alkaline phosphatase, elevated aspartate aminotransferase, diarrhea, and peripheral sensory neuropathy. These 4 patients each stopped treatment due to the AEs. No AEs exceeding grade 3 were recorded.

### Immune Monitoring

**FIGURE 2:**
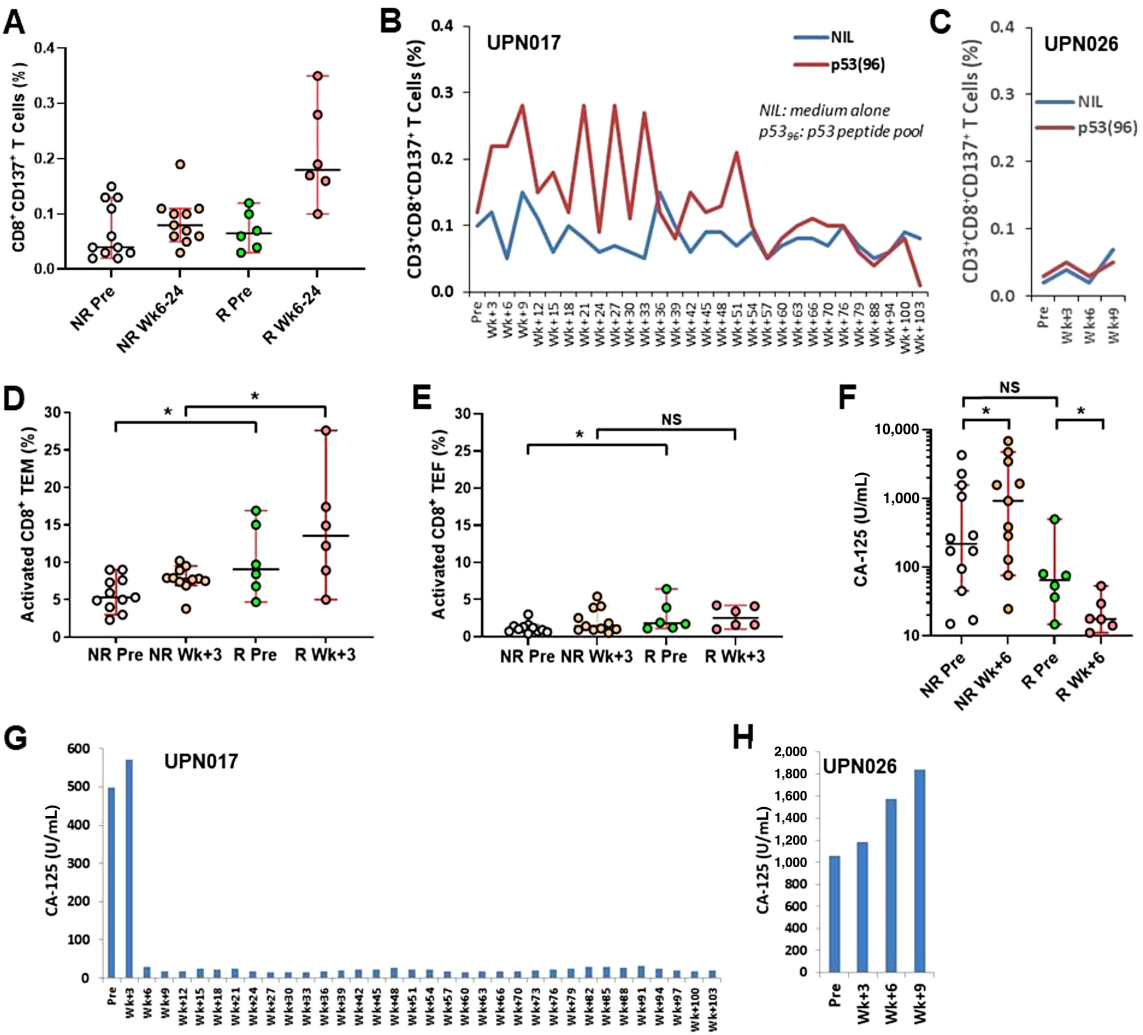
Activation of peripheral blood CD8^+^ T cells in immunologic R but not in immunologic NR during p53MVA/pembrolizumab treatment. The immune response of p53-specific CD8^+^ T cells from PBMC, as determined by flow cytometric analysis, is compiled for 18/28 evaluable in correlative studies patients (R: *n* = 6; NR: *n* = 12; **A**), as well as is shown for 1 representative R patient (**B**) and 1 NR patient (**C**). Frequencies of activated CD8^+^ TEM and TEF cells are shown in **D** and **E**, respectively. R patients normalized CA-125 serum levels to <30 U/mL by week 6 of treatment (**F** and **G**) in contrast to NR patients who exhibited gradual increase of CA-125 (**F** and **H**). *, *P < 0.05*, unpaired two-tailed *t* test except for two-tailed paired *t* test in **F** comparing NR Pre versus NR Wk+6 and R Pre versus R Wk+6 (whiskers indicate 5–95 percentiles).

Eighteen patients with ovarian cancer who underwent blood sampling at more than 3 timepoints per protocol (pretreatment, week +3 and +6, to assure sufficient timing for evaluation of immune responses to the vaccine) were deemed suitable for basic analysis in correlative studies. All clinical PR and all but one patient with clinical benefit (UPN020) were among the 18 evaluable patients. The results of the initial analysis of p53-specific responses of PBMC-derived T cells determined that 6 patients showed >2-fold (3.5-fold average, 2.0–5.8 range) increase in the frequency of CD137^+^CD8^+^ T cells above the background level. These patients were considered immunologic R. The remaining 12 patients did not generate p53-specific CD137^+^CD8^+^ T cells above background and were designated as immunologic non-responders (NR). R patients, as defined, showed increased percentages of p53_96_-specific CD137^+^CD8^+^ T cells at any timepoint between weeks 6 and 24, above the pretreatment levels as well as above the levels observed in NR (Fig. 2A). Representative results for one R UPN017 and one NR UPN026 are shown in Fig. 2B and C, respectively. The long-term PR patient UPN017 showed a distinctive increase of p53-specifc CD8^+^ T-cell percentages in response to vaccinations up to week 9, followed by fluctuating levels of p53-specifc T cells until week 51 into the treatment with gradual decline afterward (Fig. 2B). Of the 18 patients evaluable for immunologic response, there were 3 PR and 2 SD of 6 months or greater. All were within the group of immunologic R. None of the 12 immunologic NR demonstrated clinical benefit (exact test, *P* < 0.001). Similarly, the PFS was superior (log-rank *P* < 0.001) in R (*n* = 6) versus NR (*n* = 12) patients with a median PFS of 10.2 months [95% CI: 6.6-not reached (nr)] versus 1.8 months (95% CI: 1.8–nr). The response level of CD137^+^CD4^+^ T cells remained low and inconclusive.

The goal of this vaccination is to amplify a weak ongoing immune response and to recall immunologic memory for the control of disease. Proportions of activated CD8^+^ TEM cells (defined as CD3^+^CD8^+^CD197^−^CD45RO^+^CD38^+^HLA-DR^+^) in R patients increased significantly above the levels observed in NR patients within the first 3 weeks after only one cycle of p53MVA/pembrolizumab (Fig. 2D; Supplementary Fig. S1). This was not observed for TEF cells defined as CD3^+^CD8^+^CD197^−^CD45RA^+^CD38^+^HLA-DR^+^ at week 3 (Fig. 2E; Supplementary Fig. S1). It is also worth noting that pretreatment levels of TEM (Fig. 2D) and TEF cells (Fig. 2E) were higher in R than NR patients.

These early events describing CD8^+^ TEF and TEM cell dynamics correlate with CA-125 serum levels where just after two cycles of treatment by week 6, mean CA-125 significantly decreased in R patients (Fig. 2F and G) while NR patients showed gradual increase in CA-125 (Fig. 2F and H). The pretreatment levels of CA-125 in NR versus R patients are not significantly different (*P* > 0.05; Fig. 2F), suggesting that the tumor burden is not a discriminating factor between NR and R patients and both groups entered the treatment with similar status of their disease. Long-term R UPN017 patient had a transient increase of CA-125 to 570.4 U/mL on day 22 (baseline CA-125 level 498 U/mL), followed by a prolonged biochemical response (CA-125 level 29.3 U/mL on day 43 and <20 U/mL at 2 years). This patient had a corresponding radiographic PR for more than 2 years (Fig. 2G). In contrast, NR UPN026 patient exhibited progressive CA-125 increase (Fig. 2H) and parallel disease progression that resulted in just 1.8-month PFS and 5-month OS (Table 2).

### Profiling of Immune Function Gene Expression

**FIGURE 3:**
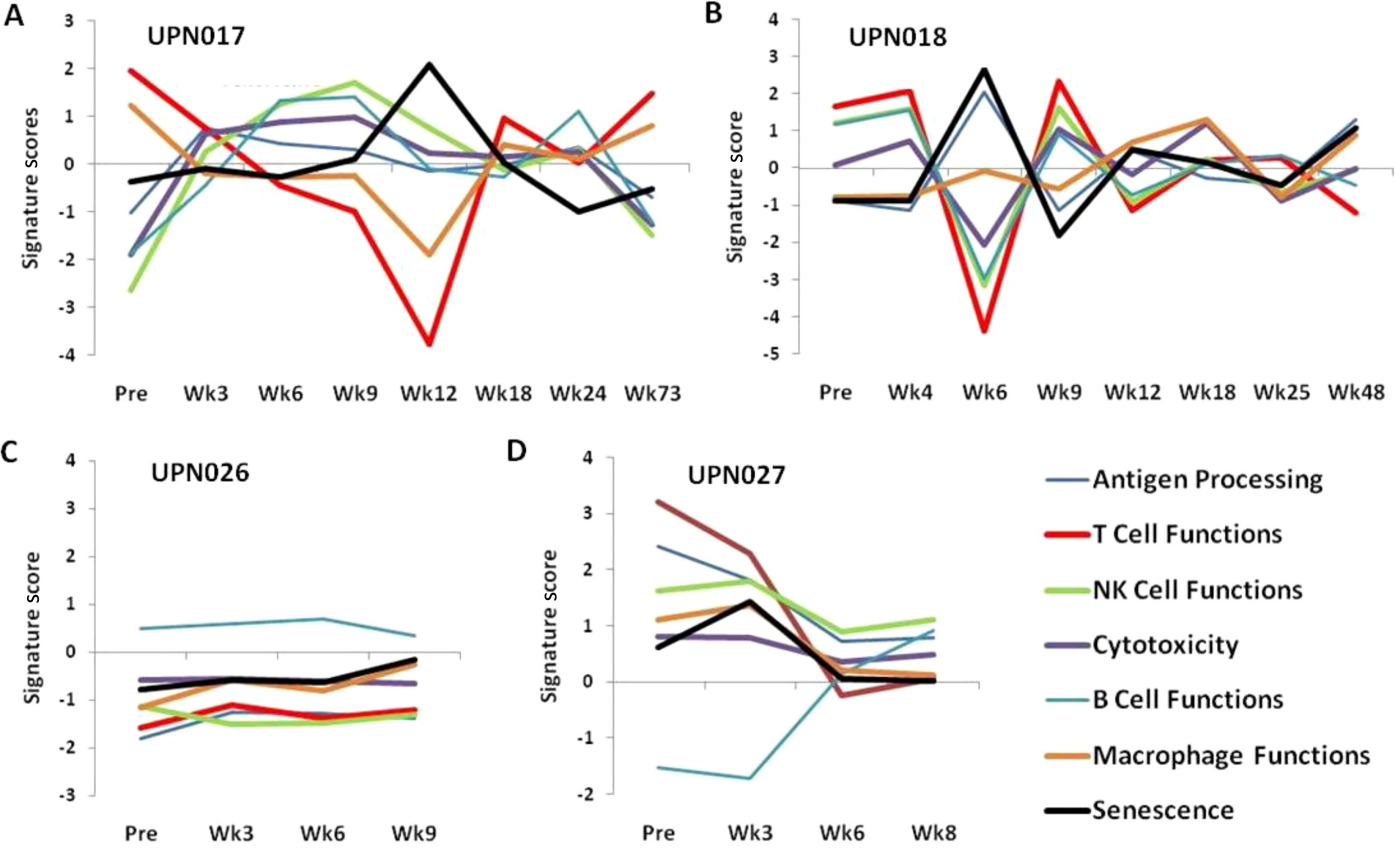
Immune function pathway scores in 2 R (**A** and **B**) and 2 NR (**C** and **D**) patients are plotted to show how they vary across time during p53MVA/pembolizumab treatment. Lines show each pathway's score of its weighted mRNA expression levels data derived from the NanoString nCounter PanCancer Immune Profiling Panel analysis. Fluctuating changes in T cell and associated immune function categories are observed in 2 R but not in 2 NR patients.

**FIGURE 4:**
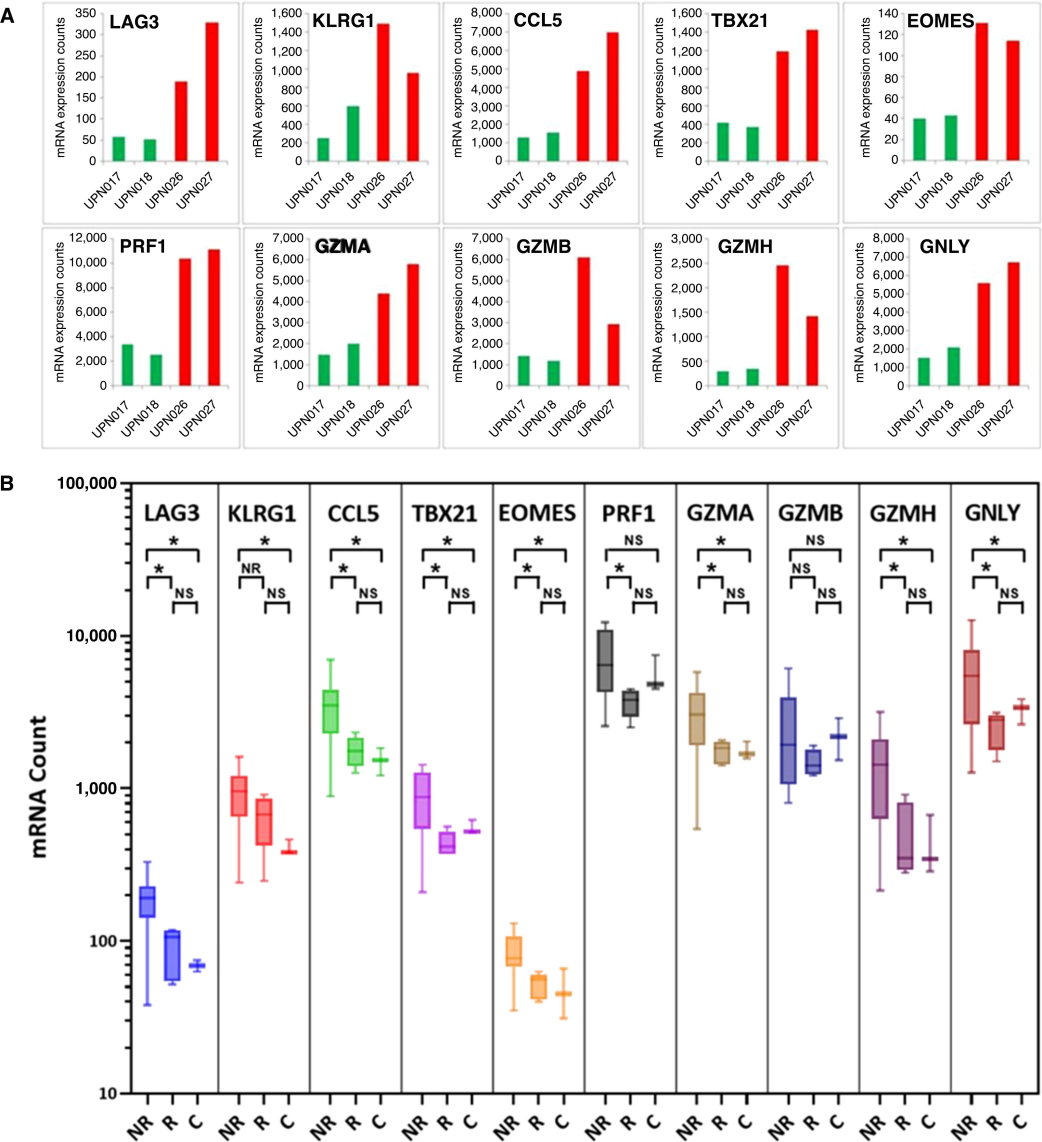
Multiplex gene expression analysis of pretreatment PBMC samples identifies a set of genes of potential predictive value. mRNA expression levels of indicated genes that differentiate R (green bars) from NR (red bars) are shown for two pairs of representative patients (**A**). Expanded analysis of mRNA expression in additional pretreatment PBMC samples in R (*n* = 5) and NR (*n* = 12), plus normal healthy controls (C, *n* = 3; **B**), confirms the trend observed in A that is further quantified in unpaired two-tailed *t* test. *, *P < 0.05*.

To enhance the scope of immune monitoring described in the section above, we further analyzed 2 R patients (UPN017 and UPN018) and 2 NR patients (UPN026 and UPN027) with multiplex gene expression analysis of PBMC samples using nCounter PanCancer Immune Profiling Panel and NanoString nCounter platform. PBMC samples from multiple timepoints during the treatment were assessed for differential expression of 730 immune profiling genes defining T-cell functions and associated immune categories. A general view of immune function pathway scores of their weighted mRNA expression calculated in nSolver software is shown in Fig. 3. Fluctuating changes in T cell and associated immune function categories occurring in three weekly intervals overlapping with timing of administration of p53MVA and/or pembrolizumab (week 0, 3, 6, 9, and more for R) were observed in 2 R (Fig. 3A and B) but not in 2 NR patients (Fig. 3C and D). These T-cell dynamics are most likely the result of reactivation of preexisting T-cell phenotypes, including TEM, in R patients during treatment. Proportions of cell subsets within PBMCs, as observed by flow cytometric and NanoString analyses, were not abnormal in any of the 28 patients and it is unlikely that the expression of genes was affected by different proportions of cells in different patients. Review of the expression levels of individual genes disclosed a set of 10 genes in pretreatment samples that distinguished 2 R (green bars) from 2 NR (red bars) patients (Fig. 4A). Expanded analysis of mRNA expression in more pretreatment PBMC samples from R (*n* = 5) and NR (*n* = 12) patients, with additional healthy controls (C; *n* = 3; Fig. 4B), confirmed the pattern observed for two pairs of patients from Fig. 4A. This pattern revealed statistically different mRNA expression levels in R versus NR as well as in C versus NR patients (Fig. 4B). There was no difference between R and C suggesting that R patients retain the immune cell/PBMC functionality expected in healthy individuals. Pre-treatment PBMC samples from NR patients, when compared with R patients and C controls, expressed higher levels of mRNA encoding LAG-3, KLRG1, CCL5 (RANTES), T-bet, Eomes (eomesodermin), perforin, granulysin, and granzymes A, B, and H.

## Discussion

KEYNOTE-100, the largest study to date of single-agent immune checkpoint for ovarian cancer, evaluated pembrolizumab in two ovarian cancer cohorts of patients with platinum-partial-sensitive, who typically have a more favorable prognosis than platinum-resistant patients. Cohort A key eligibility included 1–3 lines of prior treatment and a platinum-free interval (PFI) or treatment-free interval (TFI) of 3–12 months, and cohort B enrolled patients who received 4–6 lines of prior therapy with a PFI/TFI of ≥3 months (6). We investigated the clinical activity of combined p53MVA and pembrolizumab treatment in patients with advanced, platinum-resistant or refractory ovarian cancer, who received up to 4 lines of prior chemotherapy for recurrent disease (targeted agents or hormones not included). Patients in this report had a median number of total drug regimens of 4 (range: 2–7), compared with a median of 2 regimens (range: 1–5+) in KEYNOTE-100. And while the primary endpoint (response rate per irRECIST) for clinical efficacy in our study with a small cohort of patients was 11% (3/28 with a PR), empirically higher than the 8% response rate in KEYNOTE-100 with a more favorable patient population, this study did not achieve its predetermined threshold for success.

Our correlatives, however, provide for some insight that may both encourage and guide further study of the combination, and provide some insight for other immune therapy studies as well. In patients heavily pretreated with chemotherapy, response to vaccines are diminished. This is a well-recognized problem with influenza vaccines and more recently SARS-CoV-2 vaccines (24, 25). By selecting more heavily treated patients, we observed immune reactivity in only 6 patients, defined as a modest increase of a 2-fold increase in the frequency of CD137^+^CD8^+^ T cells above the background level. However, those 6 patients did exceptionally well. Three of the 6 patients that responded to the vaccine had a PR and 2 had SD for 6 months or longer. The median PFS for those 6 patients was 10.2 months. By comparison, in the 18 patients who were treated sufficiently to be evaluable for immunologic assessment, the 12 non-responding patients included only 1 patient with SD lasting for only 3.8 months, and the remaining 11 even progressed faster.

In addition, we note that one of the responders was progression-free at 33+ months and a patient with SD remains on single agent pembrolizumab at 28+ months. This is rare in this heavily pretreated population. While we expect that immune response as a patient characteristic is likely to select for better patients, the extent of the difference between immune responsive and immune non-responsive patients is unexpected. When a minority of patients do exceptionally well, the median PFS of the entire group is insensitive to such outliers. As a result, a vaccine that causes a documented reaction in only 21% (6/28) of the patients, is not likely to be detected as an increase in the median PFS. The signal would also be diluted in the response rate.

Combined, we note that either the combination of p53MVA with pembrolizumab has significant potential in patients who can mount an immune response or we conclude that immune response, as we define it, can predict for response to pembrolizumab or potentially similar immunotherapies. Either would represent an important advance, and would be critical to further explore. The main caveat/limitation associated with this interpretation is that the immune reactivity definition and threshold were not prespecified in the protocol, and the results are an exploratory evaluation of the biological correlatives. Further studies are required to validate these findings. Similarly, we did not observe a correlation between clinical benefit and p53 positivity by IHC, suggesting that the cutoff for positive p53 staining used in this study (≥10% of tumor cells), may not predict clinical benefit and should be re-evaluated in future studies.

Our immune monitoring studies have defined on-treatment prognostic parameters as well as, more importantly, identified pretreatment predictive markers that could facilitate selecting patients with advanced ovarian cancer that may clinically benefit from the tumor vaccine and ICB therapy. p53MVA/pembrolizumab combination therapy activated persistent p53-specific CD8^+^ T cells in the peripheral blood and this was associated with clinical benefit, and at least one long-term R demonstrated persistent CD8^+^ T-cell responsiveness to the p53_96_ peptide pool for up to 12 months. Early activation events during the first 3–6 weeks of treatment in immunologic responders resulted in the expansion of peripheral activated CD8^+^ TEM cells. Consequently, we observed a strong link between immune and clinical on-treatment responses. We have already reported on the significance of early activation of systemic immune responses and complete regression of cutaneous metastases by week 9 in a case study of a patient with triple-negative breast cancer receiving p53MVA/pembrolizumab treatment (14). High frequency of peripheral p53-specific CD8^+^ T cells was associated with lymphocytic infiltration of CD8^+^CD137^+^T cells and CD8^+^CD103^+^ T memory cells into regressing skin metastases and rapid clinical response (14). Another recently published study has shown that the TEM compartment is most sensitive to ICB treatment. Anti-PD-1 enhanced T-cell immunity during SARS-Cov-2 infection in patients with melanoma, increased expansion of TEM, and induced the appearance of activated CD38^+^HLA-DR^+^ TEM cells (26) of the same phenotype as in our study.

We have discovered an interesting pattern of expression of genes involved in immune functions. Patients responding in immune monitoring tests to the vaccine and pembrolizumab treatment displayed fluctuating mRNA expression levels linked to T cell and associated immune function pathways, such as, antigen processing, cytotoxicity, natural killer (NK), macrophage, and B-cell functions, as well as senescence. These fluctuating mRNA levels overlapped with timing of administration of p53MVA and/or pembrolizumab implying the treatment was driving the immune modulation and impacted responses. In contrast, NR patients, although monitored only for up to 9 weeks, displayed declining or stable mRNA expression in PBMC. These observations agree with published reports of increased functional complexity and transcriptional diversity of the memory CD8^+^ T-cell pool after recurrent and productive antigenic stimulation (27). In contrast, terminally differentiated CD8^+^ TEM cells do not proliferate but show high expression of effector genes and minimal expression of multipotency-associated genes (28). Continued presence of a tumor and its antigens can eventually cause T cells to lose their effector functions and become permanently dysfunctional and nonresponsive to immunotherapy (29). In our study, we have shown that immune NR before treatment expressed high levels of mRNA encoding two coinhibitory or immune checkpoint receptors LAG-3 and KLRG1, chemokine CCL5 (RANTES), T-cell and NK-cell transcription factors T-bet and Eomes, and five proteins associated with cytolytic activity of T and NK cells—perforin, granulysin, and granzymes A, B, and H. Expression of these molecules defines a signature of terminally differentiated, dysfunctional, and/or senescent CD8^+^ T cells (28, 30, 31). Dominant presence of such cells in the peripheral blood of patients with ovarian cancer strongly suggests their low potential to respond to vaccine/ICB treatment and resulting rapid disease progression.

Immune blockade receptor LAG-3 negatively regulates activation, proliferation, and homeostasis of T cells, similarly to PD-1 and CTLA-4. It also helps maintain CD8^+^ T cells in tolerogenic or exhausted state during chronic antigenic stimulation (32, 33). KLRG1 is expressed predominantly on late-differentiated TEF and TEM cells, NK cells, as well as senescent CD8^+^ T cells (34). We could speculate that the low activity of T cells in immunologically non-responding patients with ovarian cancer may be more affected by their potentially upregulated LAG-3 and KLRG1 coinhibitory/checkpoint receptors than PD-1 receptors. In further support of this interpretation is our observation that the expression of mRNA from *PDCD1* gene was very low either pretreatment or during the treatment irrespective of the clinical or immunologic status of patients. Whether this observation can affect the quality of response to anti-PD-1 would merit further investigation. CCL5 (RANTES) has already been shown to be a marker of significance in ovarian cancer. Elevated serum and ascites fluid CCL5 levels correlated with the extent of the disease and showed a potential diagnostic and prognostic value (35, 36). Cisplatin treatment induced CCL5 secretion in ovarian cancer tissue and cisplatin-resistant patients had high CCL5 levels (37). CCL5 which is produced in peripheral blood by T cells and monocytes has multiple effects on immune cells. CCL5 at high concentration may induce T-cell apoptosis (38), which could potentially compromise antitumor immune responses. T-bet and Eomes expression increases as peripheral blood T and NK cells become more mature and differentiate toward effector, memory, and eventually terminal cells (39). The relative quantity of nuclear T-bet and Eomes seems to partially define T-cell exhaustion (40). Further studies in this area may identify therapeutic opportunities resulting from controlling activity of T-bet and Eomes. Finally, we observed high expression levels of mRNA for perforin, granulysin, and granzymes A, B, and H in pretreatment PBMC samples from non-responding patients which points again at the terminal differentiated status of T cells with limited renewal capacity (28).

The choice of p53 as a tumor-associated antigen for immunotherapeutic targeting is valid for several reasons (41). *TP53* is mutated in 96% of high-grade serous carcinoma—the most frequent and the most lethal ovarian cancer. A total of 60%–70% of these mutations allow for the production of an altered p53 protein which is then overexpressed and available for processing and presentation for T-cell recognition (8). Because most mutations of the p53 involve the alteration of a single amino acid, the majority of presented epitopes are derived from nonmutated sequences. p53-specific circulating CD8^+^ T cells can be detected in some patients with cancer retaining the potential of developing anti-p53 immune responses after vaccination. Our p53MVA vaccine with its wild-type *TP53* transgene has no patient-specific mutated sequences and has the characteristics of the off-the-shelf vaccine that can function across HLA restriction barriers and does not require personalized manufacturing. In contrast, recent data in multiple tumor settings have argued for a role for individualized cancer antigens called neoantigens coupled with ICB treatment as being therapeutically superior to ICB monotherapy (42, 43). The modified vaccinia Ankara (MVA) vector itself is among the most effective activators of systemic immunity with broad T-cell repertoire and immune memory (44). Combined neoantigen and MVA therapy is being explored clinically (45).

Our study has several implications. We have established that a subset of patients with platinum-resistant, advanced ovarian cancer responds to the p53MVA and pembrolizumab therapy with prolonged PFS and OS. On the basis of this and other published studies, the p53MVA vaccine in combination with anti-PD-1 may have clinical activity benefiting a select group of patients. We acknowledge that our study did not compare combination treatment with p53MVA and anti-PD-1 or each of them singly as monotherapy. Also, evaluation of PD-L1 expression in tumor was not required in this study. However, our approach has identified potentially clinically useful predictive markers that could be developed in future studies into diagnostic tools to stratify or select evaluable patients. We postulate that the analysis of these markers in pretreatment PBMC samples may identify patients likely to respond to the p53 vaccine/pembrolizumab combination therapy or potentially pembrolizumab monotherapy. Whether this approach is the answer to the widely recognized need for identifying factors predicting clinical benefit of ICB will be evaluated in future studies.

A further implication from our study points to LAG-3 and KLRG1 checkpoint receptors that may contribute to the immunotherapy resistance in patients with ovarian cancer. These nonredundant to PD-1 immune checkpoints can potentially be targeted in combination with anti-PD-1. Merck's LAG-3–targeting antibody favezelimab has already been tested in combination with pembrolizumab in a first-in-human study in advanced colorectal cancer with promising antitumor activity (46). In addition, a fixed-dose combination of the LAG-3 blocking antibody relatlimab and the PD-1 blocking antibody nivolumab, known as opdualag (Bristol Myers Squibb) approved by the FDA, has shown a promising improvement in PFS for patients with unresectable or metastatic melanoma (47).

## Supplementary Material

Table S1 Representativeness of Study Participants

Figure S1 Gating Strategy for TEM and TEF

Table S2 Toxicities Grade 2 or Higher Grade
