# Supplementary material for: Immunologic Signatures of Peripheral Blood T Cells Reveal the Outcome of p53MVA Vaccine and Pembrolizumab Treatment in Patients with Advanced Ovarian Cancer
Source: Cancer Res Commun. 2023 Dec 20;3(12):2585–95. doi: 10.1158/2767-9764.CRC-23-0394 (PMC10732002; doi:10.1158/2767-9764.CRC-23-0394)
Supplement: Table S1 — Representativeness of Study Participants [file crc-23-0394-s01.pdf]

Supplementary Table S1. Representativeness of study participants

Cancer type(s)/subtype(s)/stage(s)/condition	Recurrent platinum resistant ovarian, fallopian tube, or primary peritoneal cancer (OC)
Considerations related to:	
Sex	OC, is a female disease. Approximately 1.1 percent of women will be diagnosed with ovarian cancer at some point during their lifetime. Five-year survival for all stages combined is 50%, but for patients with distant metastases is only 30%.
Age	This cancer mainly develops in older women. Median age at diagnosis is 63. OC rates are highest in women aged 55-64 years. Probability of dying from OC (2017-2019) at selected age intervals is the following: 0-49 years <0.1%, 50-59 – 0.1%, 60-69 years – 0.2%, 70+ years – 0.6%. The median age of death from OC is 70.
Race/ethnicity	OC average annual incidence rates per 100,000 (2016-2020) by race and ethnicity were the following: American Indian and Alaska Native – 6.9%, Non-Hispanic white – 6.7%, Non-Hispanic black – 5.7%, Hispanic – 4.9%, Asian and Pacific Islanders – 4.4%.
Geography	In the US, the number of estimated new cases in 2023 is 19,710 and estimated deaths – 13,270. In California, incidence rates for OC are similar to national average (10.9 vs 10.4 per 100,000).
Other considerations:	
Overall representativeness of this study	The age distribution of our study is similar to the average age distribution of OC in the literature, median age of 64. Our study population derived from multiethnic Southern California area was limited to our institution’s main site in Duarte, CA. Participating patient population included 10.7% Asian, 7.1% Black, 75% White, and 7.1% Unknown.
