# Supplementary material for: Immunologic Signatures of Peripheral Blood T Cells Reveal the Outcome of p53MVA Vaccine and Pembrolizumab Treatment in Patients with Advanced Ovarian Cancer
Source: Cancer Res Commun. 2023 Dec 20;3(12):2585–95. doi: 10.1158/2767-9764.CRC-23-0394 (PMC10732002; doi:10.1158/2767-9764.CRC-23-0394)
Supplement: Figure S1 — Gating Strategy for TEM and TEF [file crc-23-0394-s02.pptx]

## Slide 1
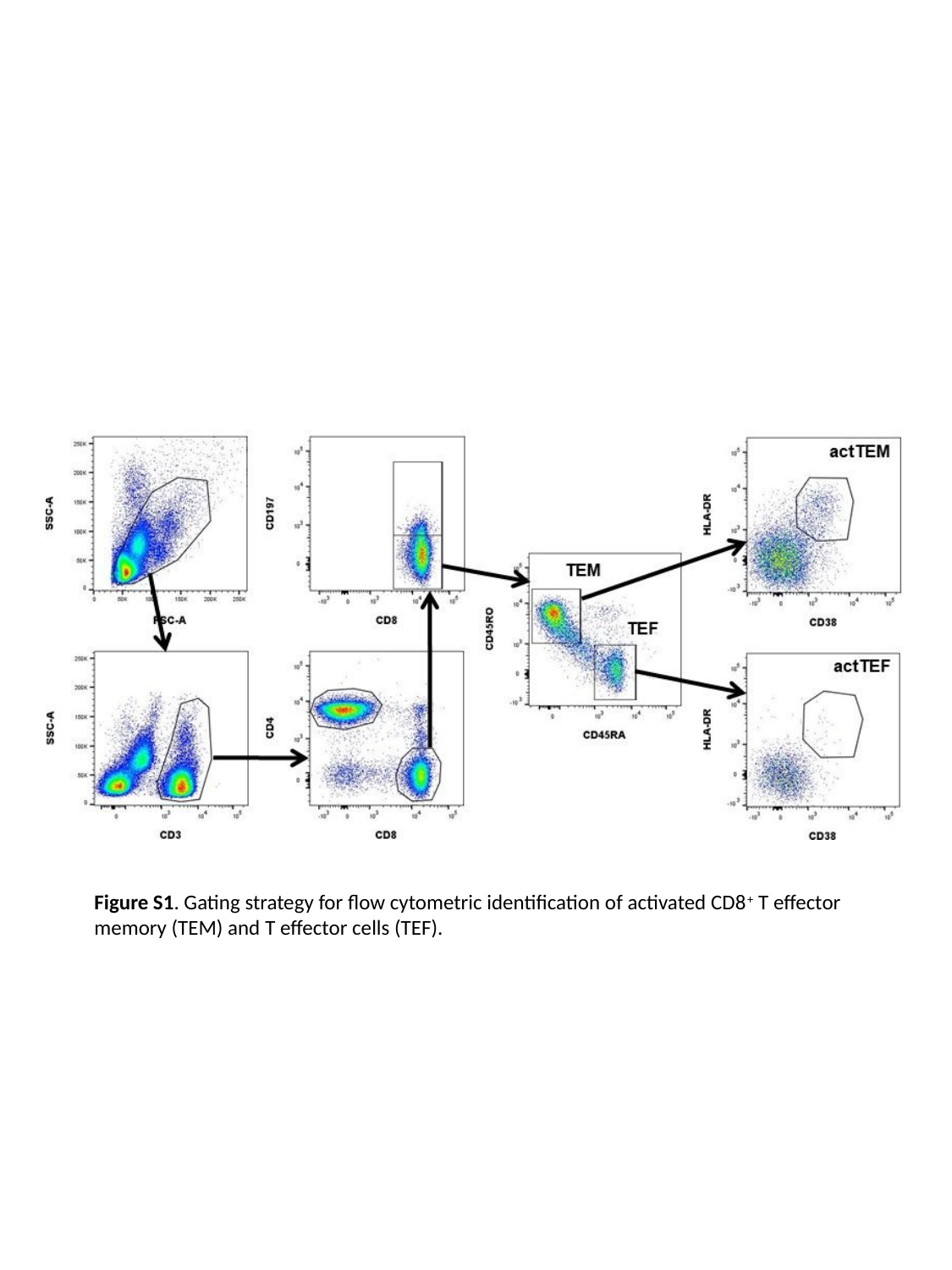

Figure S1. Gating strategy for flow cytometric identification of activated CD8+ T effector memory (TEM) and T effector cells (TEF).
