# Supplementary material for: Immunologic Signatures of Peripheral Blood T Cells Reveal the Outcome of p53MVA Vaccine and Pembrolizumab Treatment in Patients with Advanced Ovarian Cancer
Source: Cancer Res Commun. 2023 Dec 20;3(12):2585–95. doi: 10.1158/2767-9764.CRC-23-0394 (PMC10732002; doi:10.1158/2767-9764.CRC-23-0394)
Supplement: Table S2 — Toxicities Grade 2 or Higher Grade [file crc-23-0394-s03.pdf]

Supplementary Table S2. Toxicities Grade 2 or higher possibly, probably, or definitely related to treatment

Adverse Event	Grade 2	Grade 3
Diarrhea	2	1
Aspartate aminotransferase increased	1	1
Alkaline phosphatase increased		1
Peripheral sensory neuropathy		1
Fatigue	2	
Anemia	1	
Colitis	1	
Fever	1	
Flu like symptoms	1	
Adrenal insufficiency	1	
Autoimmune disorder	1	
Alanine aminotransferase increased	1	
Lymphocyte count decreased	1	
Hypoalbuminemia	1	
Hypomagnesemia	1	
Presyncope	1	
Rash maculo-papular	1	
Utricaria	1	
Hypertension	1	
Hypotension	1	
